# A multi-regional input-output table mapping China's economic outputs and interdependencies in 2012

**DOI:** 10.1038/sdata.2018.155

**Published:** 2018-08-07

**Authors:** Zhifu Mi, Jing Meng, Heran Zheng, Yuli Shan, Yi-Ming Wei, Dabo Guan

**Affiliations:** 1 The Bartlett School of Construction and Project Management, University College London, WC1E 7HB London, UK; 2 Department of Politics and International Studies, University of Cambridge, Cambridge CB3 9DT, UK; 3 Water Security Research Centre, School of International Development, University of East Anglia, Norwich NR4 7TJ, UK; 4 Center for Energy and Environmental Policy Research, School of Management and Economics, Beijing Institute of Technology, Beijing 100081, China; 5 Department of Earth System Science, Tsinghua University, Beijing 100081, China

**Keywords:** Developing world, Economics

## Abstract

Multi-regional input-output (MRIO) models are one of the most widely used approaches to analyse the economic interdependence between different regions. We utilised the latest socioeconomic datasets to compile a Chinese MRIO table for 2012 based on the modified gravity model. The MRIO table provides inter-regional and inter-sectoral economic flows among 30 economic sectors in China’s 30 regions for 2012. This is the first MRIO table to reflect China’s economic development pattern after the 2008 global financial crisis. The Chinese MRIO table can be used to analyse the production and consumption structure of provincial economies and the inter-regional trade pattern within China, as well as function as a tool for both national and regional economic planning. The Chinese MRIO table also provides a foundation for extensive research on environmental impacts by linking industrial and regional output to energy use, carbon emissions, environmental pollutants, and satellite accounts.

## Background & Summary

Although China is usually viewed as a homogenous entity in socioeconomic analysis, it is a vast country with great variations in economic development patterns, resource endowments, population density, and lifestyle. For example, the per capita gross domestic production (GDP) in Beijing, the capital of China, was more than four times the value for Gansu, a poor province in western China. China has entered a new phase of economic development since the 2008 global financial crisis – a “new normal” – in which its economic development model has changed greatly. The domestic trade patterns among different provinces might have changed because the economy is growing faster in western China than in eastern China.

Multi-regional input-output (MRIO) models are one of the most widely used approaches to analyse the economic interdependence between different regions. Because of data availability, most of the available MRIO models demonstrate inter-country economic relationships, such as the Global Trade Analysis Project (GTAP)^1^, World Input-Output Database (WIOD)^2^, Organisation for Economic Co-operation and Development Inter-Country Input-Output (OECD-ICIO)^3^, and EORA MRIO^4^. Some researchers have compiled Chinese MRIO tables based on provincial input-output tables. Zhang and Qi integrated China into eight regions and compiled MRIO tables for these eight regions for 2002 and 2007 (ref. 5). Liu *et al.* compiled MRIO table for China’s 30 provinces and 30 economic sectors for 2007 (ref. 6) and 2010^7^. The 2007 MRIO table has been used to analyse energy use^8,9^, carbon emissions^10^, air pollutants^11^ and water consumption^12,13^ embodied in trade among China’s 30 provinces. The 2010 MRIO table is the latest available version and was compiled based on the 2007 MRIO table and provincial extended input-output tables for 2010. Since only 17 Chinese provinces provide extended input-output tables for 2010, the extended input-output tables of the remaining 13 regions were compiled based on their 2007 bench-mark tables^14^. Therefore, the 2010 MRIO table is not as accurate as the 2007 MRIO table and cannot fully reflect the changes in China’s economic structure after the 2008 global financial crisis. The Chinese government released surveyed input-output tables at the provincial level for 2012. Based on these provincial input-output tables, we compiled the Chinese MRIO table for 2012 for 30 regions (excluding Hong Kong, Macao, Taiwan, and Tibet).

In the 2012 MRIO table, there are 30 economic sectors in each region. Final use is divided into five categories, including rural household consumption, urban household consumption, government consumption, fixed capital formation, and changes in inventories (Table 1). Value added is divided into four categories, including compensation of employees, net taxes on production, depreciation of fixed capital, and operating surplus (Table 2). Exports from each region are divided into international and domestic exports, and imports to each region are divided into international and domestic imports (Table 3).

**Figure 1:**
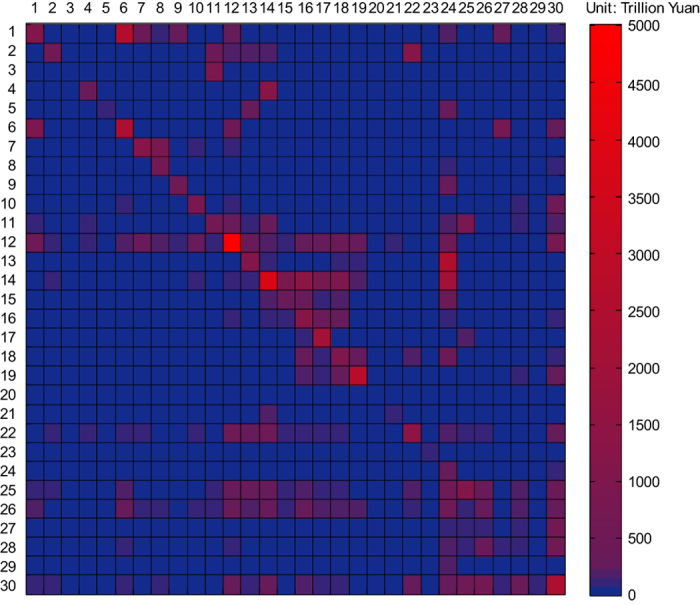
The inter-sector input-output structure among 30 Chinese economic sectors. The names of sectors 1 to 30 can be found in Table 4. The rows demonstrate the distribution of a sector’s output throughout the economy, while the columns describe the inputs required by a sector to produce its output. The colour corresponds to the inter-sector transfer, from the largest one in red to the smallest one in blue (see scale). Based on the Chinese MRIO table, we can also analyse the inter-sector transfers at the provincial level.

The Chinese MRIO table can be used to analyse provincial economies within China, as a tool for both national and regional economic planning. The table demonstrates the trade pattern among different sectors and different regions. Figure 1 demonstrates the inter-sector dependence of 30 economic sectors in China. The Chinese MRIO table can also be used to assess the economic impacts of events along supply chains and can identify economically related industry clusters. The Chinese MRIO table for 2012 can be used to estimate the changes in China’s economic development patterns by integrating the available MRIO tables for 2007 and 2010.

In addition, the Chinese MRIO table can be used to perform environmentally extended input-output analysis (EEIOA) by adding additional columns, such as energy use, carbon emissions, water consumption, and air pollutants^15,16^. For example, the data on energy inputs to each sector and each region can be applied to assess the carbon emissions embodied in the trade among 30 sectors and 30 regions. The data on China’s air pollutants can be obtained from the Multi-resolution Emission Inventory for China (MEIC)^17^. Further, the data on China’s energy consumption and carbon emissions at national and provincial levels can be downloaded freely from the China Emission Accounts and Datasets (CEADs, www.ceads.net) and are also presented in our previous paper published in Scientific Data^18^.

## Methods

We compiled an MRIO database for China’s 26 provinces and 4 cities; Hong Kong, Macao, Taiwan, and Tibet were excluded due to data unavailability. The Chinese MRIO table was compiled based on the input-output tables (IOTs) for 30 Chinese provinces that are published by the National Statistics Bureau. The IOTs demonstrate the economic linkages among 42 economic sectors at the provincial level. All provincial IOTs were aggregated into 30 sectors (see Table 4 for the concordance of sectors) because there are 30 sectors in the Chinese MRIO tables for both 2007 and 2010. We aim to build a time-series MRIO table database for China. It must be stated that the aggregation of sectors might result in bias in the input-output analysis. For example, Su and Ang^19^ indicated that sector aggregation affected the results of CO_2_ emissions embodied in trade in the environmental input–output analysis framework. In addition, Lenzen^20^ showed that both aggregation and disaggregation resulted in bias in the input-output analysis of environmental issues.

### Transfer provincial competitive IOTs into non-competitive IOTs.

IOTs can be divided into two categories according to the ways in which imports are treated, i.e., competitive and non-competitive IOTs. In competitive IOTs, imports are aggregated into a single column vector in the final use, and there is no distinction between imported input and domestically produced input. In non-competitive IOTs, the intermediate input is divided into domestic intermediate input and imported intermediate input, and the final use is divided into domestic final use and imported final use. The non-competitive IOTs are needed to compile the Chinese MRIO table. However, the original provincial IOTs are competitive IOTs.

As imports of commodities are treated as competitive imports in original provincial IOTs, the imports are also accounted for in the intermediate transactions and final demand transaction^21^. The impact of the domestic economy of an exogenous demand cannot be distinguished. It is necessary to transfer competitive imports into non-competitive imports in the compilation process. There are normally two approximation procedures to estimate the matrix of domestic transactions and interindustry imports. Method one is to assume that the layout of the matrix of competitive imports is the same as the domestic intermediate matrix, which implies that no imports are consumed directly in the final demand. Method two considers the final demand and assumes that the proportion of imports in intermediate commodities is the same as that in the final demand. In this study, we adopt the latter method by assuming that every economic sector and final use category uses imports in the same proportions^16,22^. Therefore, the matrix of competitive imports can be derived from the vector of competitive imports through multiplication by the proportion mentioned above. In the provincial competitive IOTs, the total output of a province can be expressed as

(1)O=AO+F−M

where *O* is the total output, *A* is the direct requirements matrix, *F* is the final use, and *M* is the imports. The share of import in the supply of goods to each sector is

(2)si=mioi+mi,foralli

where *s*_*i*_ is the share of import in the supply of goods to sector *i*, *o*_*i*_ is the total output of sector *i*, and *m*_*i*_ is the import of sector *i*. The new requirements matrix (*A*_*d*_) and final use (*F*_*d*_) in which only domestic goods are included are derived by

(3)Ad=diag(L−S)A

(4)Fd=diag(L−S)F

where *L* is a vector with all elements equal to 1, and diag() indicates that the vector is diagonalised. In this way, the import is removed from the intermediate use and final use and becomes a new column vector (including the import for intermediate use and final use) in the IOTs. In the new non-competitive IOTs, the total output of a province is expressed as

(5)O=AdO+Yd

### Modified gravity model to compile the MRIO

We use the gravity model and modify it with interactions among different regions for the same sector^23,24^. There are two main reasons to adopt the gravity model for estimating interregional trade flows. First, the gravity model is the most appropriate approach on the basis of available Chinese data. The approaches to construct MRIO tables can be identified as survey and non-survey approaches. The survey-based approach identifies interregional trade flows from a collection of primary data by surveys of industries and final consumers, while non-survey techniques estimate interregional trade flows from single-regional input-output tables by various modification techniques^25^. The gravity model has become the mainstream non-survey tool to estimate the interregional trade flows, not only for its simplicity, but also because of the fewer data requirements. The feasibility and reliability of this approach have been proven in many studies^26^. Other approaches are based mainly on location quotients, i.e., a type of estimation that involves scaling down. Location quotients are frequently used to estimate the interregional trade coefficients. The method is often criticised for its reliability^25^. Moreover, there are usually more data requirements for other non-survey approaches, such as the mathematical programming model developed by Canning and Wang^27^ and the computable general equilibrium (CGE) model^28^. Second, the MRIO table is also used to build a time-series MRIO table database for China. The MRIO tables for 2007 and 2010 were both constructed using the gravity model^6,7^. To maintain methodological consistency, we chose the gravity model to compile the 2012 MRIO table.

In the standard gravity model, the interregional trade flows are specified as a function of the total regional outflows, total regional inflows, and transfer cost, which is usually proxied by a distance function. The gravity model is

(6)yirs=eβ0(xirO)β1(xiOs)β2(drs)β3

where $y_{i}^{rs}$ is the trade flows of sector *i* from region *r* to region *s*, *e*^*β*0^ is the constant of proportionality, $x_{i}^{rO}$ is the total outflows of sector *i* from region *r*, $x_{i}^{Os}$ is the total inflows of sector *i* to region *s*, *d*^*rs*^ is the distance between region *r* and region *s* (we use the distance between the capital cities of the two provinces in the study), *β*_1_ and *β*_2_ are weights assigned to the masses of origin and destination, respectively, and *β*_3_ is the distance decay parameter. The above equation can be transformed into

(7)ln(yirs)=β0+β1ln(xirO)+β2ln(xiOs)−β3ln(drs)+ε

and further into

(8)Y=β0Ln+β1X1+β2X2−β3X3+ε

where *Y* is the logarithm of the trade flows of product *i* between regions, *L*_*n*_ is a vector with all elements equal to 1, *X*_1_ and *X*_2_ are the logarithm of the total outflows from origin regions and total inflows to destination regions, respectively, and *X*_3_ is the logarithm of the distance between two regions. The equation can be solved using multiple regression.

There are different interregional competition and cooperation relationships for different sectors. The industrial supply chains in some sectors are shorter, and there may be competitive relationships among different regions for these sectors, such as agriculture, food processing and textiles. In comparison, the industrial supply chains in other sectors are longer, and there may be more cooperative relationships among different regions for these sectors, such as machinery and chemicals. To reflect interregional competition and cooperation in our analysis, we introduce the concept of impact coefficients among different regions for the same sector. The impact coefficient for one sector is obtained by

(9){cigh=µig+µih|µig−µih|+minr=1,2,...,nµirg≠hcigh=1g=h

where $c_{i}^{gh}$ is the impact coefficient between regions *g* and *h* for sector *i*, ${µ}_{i}^{g}$ and ${µ}_{i}^{h}$ are the location entropy of sector *i* in regions *g* and *h*, respectively, and *n* is the number of regions. The impact coefficients indicate that stronger interactions for sector *i* occur between regions *g* and *h* if the location entropy of the sector in both regions is higher. The impact coefficient equation indicates that $c_{i}^{gh}>1$ when *g*≠*h*, and a higher value indicates stronger interactions. In addition, $c_{i}^{gh}=1$ when *g*=*h*.

We also introduce the concept of impact exponents among different regions for the same sector. It is assumed that if a larger proportion of one sector’s output is used for its own intermediate inputs, then interregional cooperation exists for the sector. The impact exponent for one sector is obtained by

(10)θi=δ¯−δi

where *θ*_*i*_ is the impact exponent for sector *i*, *δ*_*i*_ is the proportion of the total output of sector *i* that it uses as its own intermediate inputs, and $\overset{¯}{\delta }$ is the average value of *δ*_*i*_. If *θ*_*i*_>0, there are competitive relationships for sector *i*; otherwise, there are cooperative relationships for sector *i*.

We use the impact coefficients and impact exponents to modify the interregional trade flows that are obtained by the standard gravity model. The formula is

(11)Y′=Y¯/(cigh)θi

where *Y*′ represents the modified trade flows of sector *i* and $\overset{¯}{Y}$ represents the trade flows, which are obtained by the standard gravity model.

The initial trade flow matrix produced above does not meet the “double sum constraints”, in which the row and column totals match the known values in the 2012 IOTs. The RAS approach is used to adjust the trade flow matrix to ensure agreement with the summed constraints^29^. The RAS approach tends to preserve the structure of the initial matrix as much as possible with a minimum number of necessary changes to restore the row and column sums to the known values^26^.

### Adjustment according to the Chinese national IOT

In addition to the provincial IOTs, China also published a national IOT for 2012. There are great gaps between the national IOT and provincial IOTs. The sum of the total output of the 30 provinces in the provincial IOTs is 7% higher than the national total output in the national IOT. The total amount in the national IOT is assumed to be more accurate, while provincial IOTs more closely represent the economic structure at the provincial level. Therefore, we use the national IOT to adjust the total amount of output, value added, and international export and import in the MRIO, which is compiled based on provincial IOTs. Then, the adjusted MRIO table is balanced by the RAS approach.

(12)oi¯=oi∑ioi∑jojn

(13)vi¯=vi∑ivi∑jvjn

(14)ei¯=ei∑iei∑jejn

(15)mi¯=mi∑imi∑jmjn

where $\overset{¯}{o_{i}}$, $\overset{¯}{v_{i}}$, $\overset{¯}{e_{i}}$, and $\overset{¯}{m_{i}}$ are the adjusted output, value added, and international export and import for sector *i*, respectively. *o*_*i*_, *v*_*i*_, *e*_*i*_, and *m*_*i*_ are original output, value added, and international export and import for sector *i*, respectively, which are obtained from the MRIO table compiled using the modified gravity model. $o_{j}^{n}$, $v_{j}^{n}$, $e_{j}^{n}$, and $m_{j}^{n}$ are the output, value added, and international export and import for sector *i*, respectively, which are obtained from China’s national IOT.

## Data Records

The Chinese MRIO table for 2012 is stored as an excel document, and the codes are stored as a word document (Data Citation 1). The Chinese MRIO table has three main parts (Table 5). First, the top left part is a 900×900 matrix, which is the intermediate monetary flows among 30 regions and 30 sectors. Second, the top right part is a 900×150 matrix, which is the final use of 30 regions and 5 final use categories, including rural household consumption, urban household consumption, government consumption, fixed capital formation, and changes in inventories. The bottom left is a 4×900 matrix, which is the value added of 30 regions and 30 sectors. The value added is divided into compensation of employees, net taxes on production, depreciation of fixed capital, and operating surplus. In addition, international export is demonstrated as a 900×1 column vector, while international import is divided into import used as intermediate use (1×900 row vector) and import used as final use (1×150 row vector). The total output column vector is equal to the transposition of the total input row vector.

## Technical Validation

The Chinese MRIO table is compiled using the modified gravity model. The multiple regression impacts the quality of the MRIO table. The regression results for 30 economic sectors are shown in Table 6. It can be observed that the goodness of fit (*R*^2^) for most sectors is greater than 0.4, except for metal mining and petroleum and gas. The *R*^2^ value for the textile sector exceeds 0.8.

The RAS approach is used to adjust the trade flow matrix to ensure agreement with the “double sum constraints”. There is a 900×1 column vector that reflects the balance error in the Chinese MRIO table. The balance error in the table is caused mainly by the balance error in the provincial IO tables and the gap between total inflows and outflows at the provincial level. The proportions of error in the total output for most sectors are within ±5%, which is close to the values in the Chinese MRIO tables for 2007 and 2010 (refs 6,7).

China also published a national single-region input-output (SRIO) table for 2012 in addition to the provincial IOTs. We compared the sector dependence between the MRIO and SRIO tables (Table 7). It can be observed that the proportions of other sectors' input relative to the total intermediate input for each sector are similar in the two tables. Most of the differences are within ±15%. The largest difference is 22%, i.e., for gas and water production and supply.

**Figure 2:**
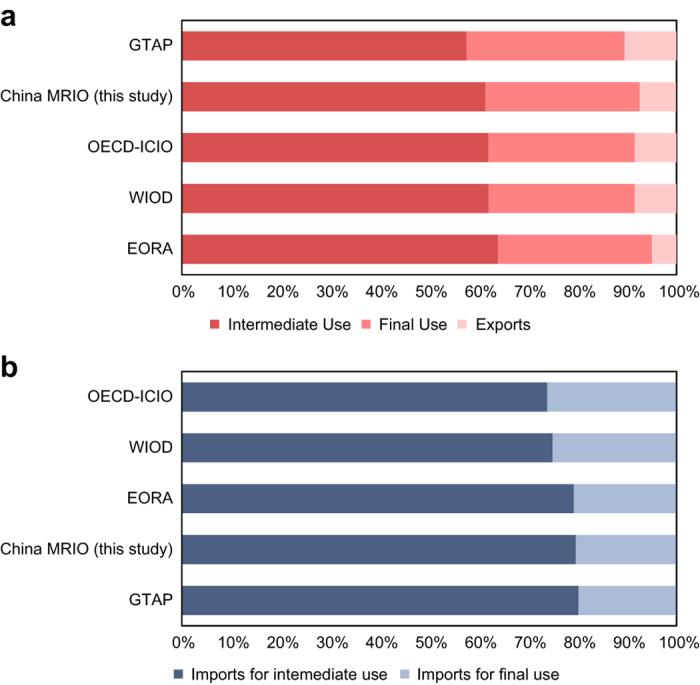
Comparisons between the Chinese MRIO table and other global MRIO tables. (**a**) compares the structure of intermediate use, final use, and exports. (**b**) compares the structure of imports for intermediate and final use. The intermediate use and final use exclude imports, so the summation of intermediate use, final use, and exports is equal to the total output. Data sources: Global Trade Analysis Project (GTAP)^1^, World Input-Output Database (WIOD)^2^, Organisation for Economic Co-operation and Development Inter-Country Input-Output (OECD-ICIO)^3^, and EORA^4^.

The structure of intermediate use, final use, exports, imports, and output is critical for the quality of the MRIO table. We compared the structure of the Chinese MRIO table and other four widely used global MRIO tables that include China, i.e., the Global Trade Analysis Project (GTAP)^1^, World Input-Output Database (WIOD)^2^, Organisation for Economic Co-operation and Development Inter-Country Input-Output (OECD-ICIO)^3^, and EORA^4^. With respect to the Chinese MRIO table, the proportions of intermediate use, final use, and export in the total output are 61, 31, and 7%, respectively. The largest proportion of intermediate use is 64% in EORA, while the smallest proportion is 58% in GTAP (Fig. 2a). In the Chinese MRIO table, 79.6% of China’s imports are used for intermediate use, while the remaining 20.4% are used for final use. The largest proportion of imports for intermediate use is 80.2% in GTAP, while the smallest proportion is 73.9% in OECD-ICIO (Fig. 2b).

## Additional information

**How to cite this article**: Mi, Z. *et al*. A multi-regional input-output table mapping China's economic outputs and interdependencies in 2012. *Sci. Data* 5:180155 doi: 10.1038/sdata.2018.155 (2018).

**Publisher’s note**: Springer Nature remains neutral with regard to jurisdictional claims in published maps and institutional affiliations.

## Supplementary Material



## Figures and Tables

**Table 1:** Final use for 30 Chinese regions in 2012 (in billion Chinese Yuan).

**No.**	**Region**	**Rural household consumption**	**Urban household consumption**	**Government consumption**	**Fixed capital formation**	**Inventory increase**	**Total final use**
1	Beijing	37	512	394	622	33	1,598
2	Tianjin	28	254	150	824	47	1,303
3	Hebei	199	491	290	1,335	14	2,329
4	Shanxi	102	243	142	678	50	1,215
5	Inner Mongolia	70	226	162	1,087	40	1,585
6	Liaoning	118	580	193	1,329	36	2,256
7	Jilin	78	221	139	808	11	1,257
8	Heilongjiang	95	299	249	692	28	1,363
9	Shanghai	41	730	248	620	59	1,699
10	Jiangsu	395	1,050	648	1,811	83	3,988
11	Zhejiang	225	844	330	1,235	65	2,699
12	Anhui	161	393	188	748	57	1,547
13	Fujian	130	403	164	909	92	1,698
14	Jiangxi	136	284	138	558	19	1,135
15	Shandong	339	951	553	2,256	135	4,234
16	Henan	273	590	317	1,917	35	3,132
17	Hubei	162	465	256	1,063	49	1,996
18	Hunan	202	485	212	1,061	44	2,005
19	Guangdong	276	1,761	552	1,950	74	4,613
20	Guangxi	128	308	143	788	46	1,412
21	Hainan	23	60	40	172	5	300
22	Chongqing	66	289	123	535	27	1,038
23	Sichuan	288	517	251	1,070	35	2,161
24	Guizhou	88	170	92	360	9	718
25	Yunnan	144	258	156	703	55	1,317
26	Shaanxi	99	297	174	858	17	1,445
27	Gansu	72	132	95	282	26	606
28	Qinghai	16	36	36	168	-16	240
29	Ningxia	19	55	33	115	10	232
30	Xinjiang	60	150	167	485	28	889

**Table 2:** Value added for 30 Chinese regions in 2012 (in billion Chinese Yuan).

**No.**	**Region**	**Compensation of employees**	**Net taxes on production**	**Depreciation of fixed capital**	**Operating surplus**	**Total value added**
1	Beijing	832	270	211	335	1,648
2	Tianjin	465	197	139	388	1,189
3	Hebei	1,259	314	309	568	2,450
4	Shanxi	490	183	172	271	1,117
5	Inner Mongolia	665	141	157	547	1,509
6	Liaoning	1,092	423	353	428	2,295
7	Jilin	423	171	184	323	1,101
8	Heilongjiang	499	194	143	430	1,266
9	Shanghai	773	371	227	490	1,861
10	Jiangsu	2,343	581	836	1,770	5,529
11	Zhejiang	1,362	494	353	1,102	3,311
12	Anhui	717	301	175	394	1,587
13	Fujian	920	259	195	442	1,816
14	Jiangxi	404	210	102	478	1,194
15	Shandong	1,605	729	531	1,748	4,612
16	Henan	1,337	346	316	730	2,729
17	Hubei	1,042	230	269	526	2,067
18	Hunan	1,012	304	220	507	2,042
19	Guangdong	2,511	681	695	1,227	5,113
20	Guangxi	662	168	126	245	1,202
21	Hainan	134	50	39	40	263
22	Chongqing	470	161	115	307	1,052
23	Sichuan	1,081	221	305	594	2,201
24	Guizhou	346	71	88	127	632
25	Yunnan	483	203	107	163	956
26	Shaanxi	574	251	133	375	1,333
27	Gansu	277	81	72	92	522
28	Qinghai	76	26	31	42	175
29	Ningxia	105	40	35	37	216
30	Xinjiang	421	76	96	99	692

**Table 3:** Exports and imports for 30 Chinese regions in 2012 (in billion Chinese Yuan).

**No.**	**Region**	**Exports to other provinces**	**Exports to other countries**	**Imports from other provinces**	**Imports from other countries**
1	Beijing	1,723	401	1,513	659
2	Tianjin	774	273	828	364
3	Hebei	1,372	184	1,237	154
4	Shanxi	587	36	651	67
5	Inner Mongolia	993	261	1,039	302
6	Liaoning	1,320	300	1,262	325
7	Jilin	506	32	602	103
8	Heilongjiang	727	50	792	90
9	Shanghai	1,805	969	1,515	1,224
10	Jiangsu	3,143	1,844	2,336	1,043
11	Zhejiang	1,357	1,333	1,433	542
12	Anhui	1,515	132	1,527	60
13	Fujian	507	516	194	783
14	Jiangxi	556	101	520	54
15	Shandong	894	1,165	709	914
16	Henan	1,803	130	2,162	146
17	Hubei	251	209	184	222
18	Hunan	832	47	785	48
19	Guangdong	1,445	3,186	1,729	2,539
20	Guangxi	408	79	590	123
21	Hainan	272	14	265	74
22	Chongqing	811	9	804	8
23	Sichuan	369	195	383	124
24	Guizhou	313	21	405	12
25	Yunnan	403	16	744	48
26	Shaanxi	959	174	1,097	153
27	Gansu	325	13	380	49
28	Qinghai	40	6	98	17
29	Ningxia	126	8	143	5
30	Xinjiang	367	41	575	27

**Table 4:** Concordance of sectors for provincial IOTs and the Chinese MRIO table.

**No.**	**Sectors for the Chinese MRIO table**	**Sectors for provincial IOTs**
1	Agriculture	Agriculture
2	Coal mining	Coal mining
3	Petroleum and gas	Petroleum and gas
4	Metal mining	Metal mining
5	Nonmetal mining	Nonmetal mining
6	Food processing and tobacco	Food processing and tobacco
7	Textiles	Textiles
8	Clothing, leather, fur, etc.	Clothing, leather, fur, etc.
9	Wood processing and furnishing	Wood processing and furnishing
10	Paper making, printing, stationery, etc.	Paper making, printing, stationery, etc.
11	Petroleum refining, coking, etc.	Petroleum refining, coking, etc.
12	Chemical industry	Chemical industry
13	Nonmetal products	Nonmetal products
14	Metallurgy	Metallurgy
15	Metal products	Metal products
16	General and specialist machinery	General machinery
		Specialist machinery
17	Transport equipment	Transport equipment
18	Electrical equipment	Electrical equipment
19	Electronic equipment	Electronic equipment
20	Instrument and meter	Instrument and meter
21	Other manufacturing	Other manufacturing
		Waster and flotsam
		Repair service for metal products, machinery and equipment
22	Electricity and hot water production and supply	Electricity and hot water production and supply
23	Gas and water production and supply	Gas production and supply
		Water production and supply
24	Construction	Construction
25	Transport and storage	Transport and storage
26	Wholesale and retail	Wholesale and retail
27	Hotel and restaurant	Hotel and restaurant
28	Leasing and commercial services	Leasing and commercial services
29	Scientific research	Scientific research
30	Other services	Information transfer and software
		Banking
		Real estate trade
		Management of water conservancy, environment and public establishments
		Residential services and other services
		Education
		Sanitation and social welfare
		Culture, sports and entertainment
		Public management and social organisations

**Table 5:** The structure of the Chinese multi-regional input-output table.

**Output (right)**	**Input (down)**						**Intermediate use**				**Final use**			**Others**	**Total output**						
	**Region 1**	**…**					**Region 30**			**Total intermediate use**	**Region 1**					**…**	**Region 30**			**Exports**	**Total final use**
	**Sector 1**	**…**	**Sector 30**	**…**	**Sector 1**	**…**	**Sector 30**		**Consumption**	**Capital formation**	**Inventory increase**	**…**	**Consumption**			**Capital formation**	**Inventory increase**				
Intermediate input	Region 1	Sector 1	Z_1,1_	…			Z_1,30_			TIU	Y_1,1_			…	Y_1,30_			E_1_	TFU	O_1_	X_1_
		…																			
		Sector 30																			
	…	…	…	…			…					…			…	…				…	…	…
	Region 30	Sector 1	Z_30,1_	…			Z_30,30_					Y_30,1_			…	Y_30,30_				E_30_	O_30_	X_30_
		…																				
		Sector 30																				
	Imports						I_inter,1_				…	I_inter,30_				I_final,1_			…	I_final,30_	0	0	0	0
	Total intermediate inputs						TII												
Value added	Compensation of employees						V_1,1_			…	V_1,30_						
	Net taxes on production						V_2,1_			…	V_2,30_		
	Depreciation of fixed capital						V_3,1_			…	V_3,30_		
	Operating surplus						V_4,1_			…	V_4,30_		
	Total value added						TVA			
Total input							X_1_^T^			…	X_30_^T^		
The names of regions 1 to 30 and sectors 1 to 30 can be found in Table 1 and Table 4, respectively. Z_i, j_ is the intermediate monetary flows from region i to region j. Y_i, j_ is region j’s use of products produced in region i during their final use. V_1,j_, V_2,j_, V_3,j_, and V_4,j_ are the compensation of employees, net taxes on production, depreciation of fixed capital, and operating surplus, respectively, of region j. E_i_ is the export of region i, O_i_ is the balance error of region i, X_i_ is the total output of region i, and X_i_^T^ is the total input of region j. I_inter, j_ is the import used as in intermediate use of region j, and I_final, j_ is the import used in the final use of region j. TIU is the total intermediate use, TFU is the total final use, TII is the total intermediate input, and TVA is total value added. For all variables, i=1, 2,…, 30 and j=1, 2,…, 30. Consumption is further divided into rural household consumption, urban household consumption and government consumption.																					

**Table 6:** The regression results for the 30 economic sectors in the gravity model.

**No.**	**Sectors**	**β_0_**	**β_1_**	**β_2_**	**β_3_**	**R^2^**
1	Agriculture	−7.03	0.96	0.58	−1.17	0.56
2	Coal mining	2.99	0.31	0.46	−1.55	0.42
3	Petroleum and gas	0.10	0.18	0.22	−0.67	0.12
4	Metal mining	1.67	0.39	0.48	−1.22	0.43
5	Nonmetal mining	−3.09	0.38	0.75	−1.10	0.49
6	Food processing and tobacco	−7.48	0.94	0.60	−1.17	0.48
7	Textiles	−11.42	0.78	1.10	−0.86	0.83
8	Clothing, leather, fur, etc.	−7.29	0.83	0.67	−1.11	0.71
9	Wood processing and furnishing	−1.81	0.62	0.55	−1.12	0.56
10	Paper making, printing, stationery, etc.	−9.70	0.59	1.14	−1.13	0.52
11	Petroleum refining, coking, etc.	−12.13	0.67	1.07	−0.94	0.62
12	Chemical industry	−7.75	0.94	0.66	−1.07	0.73
13	Nonmetal products	−5.97	0.95	0.60	−1.30	0.55
14	Metallurgy	−14.60	0.71	1.31	−1.01	0.76
15	Metal products	−0.77	0.61	0.47	−1.27	0.67
16	General and specialist machinery	−8.58	0.69	0.90	−1.13	0.72
17	Transport equipment	−6.01	1.01	0.47	−1.28	0.75
18	Electrical equipment	−10.68	0.78	0.98	−1.20	0.74
19	Electronic equipment	−15.41	0.67	1.43	−1.20	0.67
20	Instrument and meter	1.51	0.54	0.28	−1.16	0.55
21	Other manufacturing	−12.01	0.69	1.11	−0.93	0.69
22	Electricity and hot water production and supply	10.89	0.20	0.18	−1.92	0.40
23	Gas and water production and supply	6.05	0.18	0.25	−1.29	0.43
24	Construction	9.55	0.06	0.05	−1.45	0.49
25	Transport and storage	−1.29	0.54	0.52	−0.95	0.63
26	Wholesale and retailing	1.12	0.41	0.36	−1.07	0.57
27	Hotel and restaurant	3.37	0.23	0.26	−0.99	0.45
28	Leasing and commercial services	5.02	0.39	0.22	−1.21	0.55
29	Scientific research	8.33	0.06	0.00	−1.25	0.43
30	Other services	−3.90	0.66	0.59	−0.94	0.79
*β*0, *β*1, *β*2, and *β*3 are regression coefficients. *β*1, *β*2 are weights assigned to the masses of origin and destination, respectively, and *β*_3_ is the distance decay parameter. *R*^2^ is the goodness of fit.						

**Table 7:** Proportions of other sectors' input in the total intermediate input in the Chinese MRIO and SRIO tables.

**No.**	**Sectors**	**MRIO table**	**SRIO table**	**Differences**
1	Agriculture	67%	67%	0%
2	Coal mining	64%	68%	4%
3	Petroleum and gas	87%	98%	11%
4	Metal mining	68%	77%	9%
5	Nonmetal mining	83%	98%	15%
6	Food processing and tobacco	60%	70%	10%
7	Textiles	49%	49%	0%
8	Clothing, leather, fur, etc.	70%	83%	13%
9	Wood processing and furnishing	60%	56%	−4%
10	Paper making, printing, stationery, etc.	62%	66%	4%
11	Petroleum refining, coking, etc.	76%	91%	15%
12	Chemical industry	41%	46%	5%
13	Nonmetal products	69%	73%	4%
14	Metallurgy	55%	57%	2%
15	Metal products	83%	83%	0%
16	General and specialist machinery	75%	72%	−3%
17	Transport equipment	54%	61%	7%
18	Electrical equipment	75%	81%	6%
19	Electronic equipment	36%	40%	4%
20	Instrument and meter	87%	82%	−5%
21	Other manufacturing	85%	93%	8%
22	Electricity and hot water production and supply	61%	56%	−5%
23	Gas and water production and supply	66%	88%	22%
24	Construction	98%	96%	−2%
25	Transport and storage	70%	91%	21%
26	Wholesale and retailing	88%	77%	−11%
27	Hotel and restaurant	99%	100%	1%
28	Leasing and commercial services	91%	77%	−14%
29	Scientific research	90%	85%	−5%
30	Other services	68%	82%	14%
